# Failure of pharmacological DVT prophylaxis due to cold chain disruption: a six-month audit from a tertiary surgical ward in Cairo

**DOI:** 10.1186/s12893-025-03326-5

**Published:** 2025-12-17

**Authors:** Ahmed Shafik, Haytham Elessawy, Youssef Karkoucha Gobrial, Abdelrahman Lotfy

**Affiliations:** 1 Department of Surgery, New Giza University (NGU), Cairo, Egypt; 2 New Giza University (NGU), Cairo, Egypt; 3 Department of General Surgery, Faculty of Medicine, Cairo University, Cairo, Egypt

**Keywords:** Deep vein thrombosis, Audit, Surgery, Cold chain, Enoxaparin, Patient safety

## Abstract

**Background:**

Deep vein thrombosis (DVT) remains a major postoperative complication despite routine prophylaxis. This audit investigated an unexpected rise in DVT incidence in a tertiary surgical ward in Cairo, Egypt.

**Methods:**

A six-month retrospective audit (December 2024–June 2025) included 212 adult patients undergoing general surgical procedures. All received enoxaparin prophylaxis. Clinically suspected DVTs were identified using Wells Score criteria and confirmed by duplex Doppler ultrasonography. Cold chain integrity of enoxaparin storage was reviewed in collaboration with pharmacy and biomedical engineering teams. Descriptive and comparative analyses were performed.

**Results:**

Eighteen patients (8.5%) were clinically suspected to have DVT within 30 days postoperatively; 14 cases (6.6%) were confirmed by Doppler. Nine occurred during index admission and five during follow-up. Eleven of 14 confirmed cases (78.6%) were temporally linked to a malfunctioning ward refrigerator with storage temperatures exceeding 25 °C. Patients with DVT were more likely to have undergone emergency surgery and less likely to have received mechanical prophylaxis, although these differences were not statistically significant. No major bleeding events were systematically recorded. Patients with DVT were more likely to have undergone emergency surgery and less likely to have received mechanical prophylaxis, although these differences were not statistically significant. By contrast, exposure to enoxaparin from the malfunctioning refrigerator was significantly associated with postoperative DVT (*p* = 0.002).

**Conclusion:**

Cold chain failure was strongly associated with prophylaxis failure and is the most plausible contributor, though definitive pharmacological degradation was not confirmed. Infrastructure monitoring and mechanical prophylaxis adherence are essential to effective thromboprophylaxis. A prospective re-audit is scheduled following corrective measures.

## Introduction

 Venous thromboembolism (VTE), encompassing deep vein thrombosis (DVT) and pulmonary embolism (PE), is a significant cause of postoperative morbidity and mortality [1, 2]. International guidelines such as those from NICE and the American College of Chest Physicians (ACCP) provide clear recommendations for VTE prophylaxis in surgical patients, emphasizing both pharmacological and mechanical strategies [3].

At Kasr Al Ainy’s General Surgery Department, pharmacological prophylaxis is routinely administered postoperatively using low molecular weight heparin (LMWH), stored and dispensed under cold-chain conditions [4]. However, during routine postoperative review meetings, a rising trend in clinically diagnosed DVT cases was noted over a six-month period. This prompted a departmental audit to evaluate our compliance with VTE prophylaxis protocols and investigate potential systemic failures.

Surprisingly, the audit revealed that despite documented adherence to guidelines, the rate of postoperative DVT remained unacceptably high. Further investigation identified a critical failure in cold storage conditions for LMWH, raising suspicion of compromised drug stability and efficacy.

This audit explores the extent of the issue, evaluates adherence to prophylaxis protocols, and highlights the importance of infrastructure reliability in achieving guideline-compliant outcomes. To our knowledge, this represents one of the first documented audits linking pharmacological thromboprophylaxis failure to cold chain malfunction in a tertiary surgical setting.

## Methods

### Study design and setting

This was a retrospective clinical audit conducted in the general surgical wards of Kasr Al Ainy Teaching Hospital, Cairo University, Egypt, covering the six-month period from 1 December 2024 to 1 June 2025. The audit evaluated compliance with international venous thromboembolism (VTE) prophylaxis guidelines and investigated factors contributing to an unexpectedly high incidence of postoperative deep vein thrombosis (DVT).

### Patients

All consecutive adult patients (≥ 18 years) admitted for elective or emergency general surgical procedures during the audit period were included. Exclusion criteria were: (1) pre-existing DVT or pulmonary embolism at the time of admission, (2) therapeutic anticoagulation for other indications, or (3) incomplete records regarding perioperative prophylaxis.

### DVT diagnosis

Clinically suspected DVT was defined according to standard clinical features (unilateral limb swelling, pain, erythema, unexplained fever, or unexplained tachycardia) and assessed in line with the Wells Score framework [5]. All suspected cases were referred for duplex Doppler ultrasonography performed by the radiology department, which served as the confirmatory diagnostic test. The primary outcome was the incidence of radiologically confirmed postoperative DVT within 30 days of surgery. Secondary outcomes included the incidence of clinically suspected but unconfirmed cases, and exploratory evaluation of potential systemic contributors to prophylaxis failure, including cold chain integrity and adherence to mechanical prophylaxis.

### Data collection

Patient records were reviewed for demographic details, surgical urgency (elective vs. emergency), procedure type, perioperative prophylaxis (drug, dose, timing), and use of mechanical prophylaxis (graduated compression stockings or intermittent pneumatic compression). Records of the hospital’s pharmacy and ward medication refrigerators were audited in collaboration with biomedical engineering and pharmacy teams to identify potential breaches in cold chain storage of low molecular weight heparin (LMWH, enoxaparin/Clexane^®^). Drug storage temperatures were compared against manufacturer recommendations (2–8 °C). Any documented refrigeration malfunctions were correlated with the timing of DVT cases.

### Statistical analysis

Descriptive statistics were used to summarize patient characteristics and outcomes. Continuous variables are presented as mean ± standard deviation (SD) or median with interquartile range (IQR), depending on distribution. Categorical variables are expressed as frequencies and percentages. Patients with confirmed DVT were compared to those without DVT using chi-square or Fisher’s exact test for categorical variables, and t-test or Mann–Whitney U test for continuous variables. A p-value < 0.05 was considered statistically significant. Analyses were performed using IBM SPSS Statistics (version 28.0).

### Re-audit plan

In line with the audit cycle, interventions to improve cold chain reliability and compliance with mechanical prophylaxis were implemented from June 2025. A prospective re-audit is scheduled for August 2025 to assess changes in postoperative DVT incidence following these corrective measures.

## Results

### Patient characteristics

A total of 212 adult patients underwent general surgical procedures during the six-month audit period (December 2024 – June 2025). The cohort had a median age of 56 years (IQR 45–64), and 124 patients (58.5%) were male. Emergency operations accounted for 92 cases (43.4%), while 120 (56.6%) were elective. Mechanical prophylaxis (graduated compression stockings or intermittent pneumatic compression) was applied in 94 patients (44.3%). All patients received pharmacological prophylaxis with enoxaparin (Clexane^®^) according to institutional protocols. Table 1 summarizes the weekly distribution of DVT cases and corresponding refrigerator temperature logs recorded during the six-month audit period.

**Table 1:** Weekly DVT cases and temperature breaches

Week	DVT Cases	Temperature Breaches
Week 1	0	0
Week 2	1	1
Week 3	0	0
Week 4	1	2
Week 5	2	2
Week 6	1	1
Week 7	0	0
Week 8	1	2
Week 9	0	0
Week 10	0	0
Week 11	1	1
Week 12	2	3
Week 13	0	0
Week 14	1	1
Week 15	1	1
Week 16	0	0
Week 17	0	0
Week 18	1	2
Week 19	0	0
Week 20	2	3
Week 21	1	2
Week 22	0	0
Week 23	1	1
Week 24	0	0
Week 25	1	1
Week 26	1	1

### Incidence of DVT

Eighteen patients (8.5%) were clinically suspected to have developed DVT within 30 days postoperatively. Of these, 14 cases (6.6%) were confirmed by duplex Doppler ultrasonography and were considered the primary outcome events. The remaining four patients were lost to confirmatory imaging after initial suspicion, largely due to discharge before testing; these cases are reported separately as suspected but unconfirmed events.

**Fig. 1:**
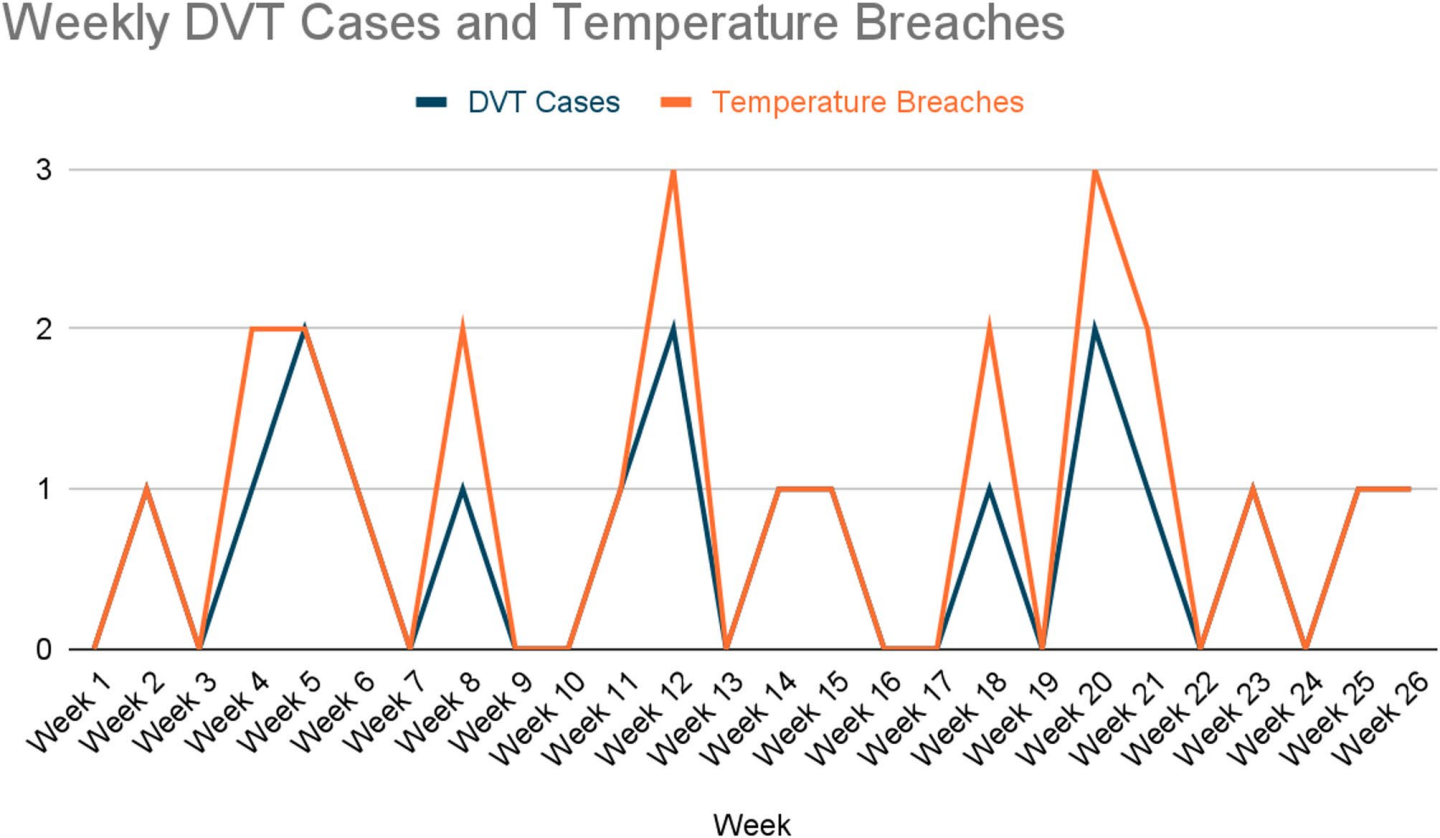
Temporal correlation between DVT cases and documented refrigerator temperature breaches over the 26-week audit period

Of the 14 confirmed cases, 9 (64.3%) were diagnosed during the index admission and 5 (35.7%) were diagnosed within 30 days of discharge during routine follow-up visits. A temporal correlation between weekly DVT cases and refrigerator temperature breaches is illustrated in Fig. 1.

Patients with DVT were older, more likely to undergo emergency surgery, and less likely to receive mechanical prophylaxis, although differences did not reach statistical significance. By contrast, enoxaparin exposure from the malfunctioning refrigerator was significantly associated with postoperative DVT (*p* = 0.002). Baseline characteristics of patients with and without confirmed DVT are summarized (Table 2).

**Table 2:** Baseline characteristics of patients with and without confirmed postoperative DVT

Variable	DVT (*n* = 14)	No DVT (*n* = 198)	*p*-value
Age, median (IQR), years	59 (53–67)	55 (44–63)	0.28
Male sex, n (%)	9 (64.3%)	113 (57.1%)	0.62
Emergency surgery, n (%)	8 (57.1%)	84 (42.4%)	0.32
Elective surgery, n (%)	6 (42.9%)	114 (57.6%)	–
Mechanical prophylaxis applied, n (%)	4 (28.6%)	90 (45.5%)	0.21
Enoxaparin from affected fridge, n (%)	11 (78.6%)	62 (31.3%)	0.002*

Statistical testing performed using chi-square or Fisher’s exact test as appropriate for categorical variables, and Mann-Whitney U test for continuous variables (age)

*p < 0.05 considered statistically significant

### Comparative analysis

Patients who developed DVT were more likely to have undergone emergency surgery and less likely to have received mechanical prophylaxis compared to those without DVT. No statistically significant differences in age or sex were observed. Due to the limited sample size, these comparisons should be interpreted with caution.

### Bleeding events

No major bleeding complications, re-operations for haemorrhage, or transfusion events were systematically recorded during the audit period. This represents a limitation of the dataset.

## Discussion

This audit highlights a critical systems failure—temperature-dependent degradation of low molecular weight heparin (LMWH) due to cold chain malfunction—as a plausible explanation for an unexpectedly high incidence of postoperative deep vein thrombosis (DVT) in a tertiary surgical ward. Despite adherence to international pharmacological prophylaxis protocols, 6.6% of patients developed radiologically confirmed DVTs, a rate substantially higher than the 1–2% reported in contemporary literature [6, 7], including regional audits [7]. This discrepancy underscores the importance of infrastructure reliability in ensuring the effectiveness of evidence-based thromboprophylaxis. This provides new evidence underscoring the critical role of drug storage integrity as a systems-level determinant of surgical patient safety. Notably, no formal baseline audit was available for comparison, but departmental estimates suggest historical postoperative DVT rates were consistently below 2%, in line with international benchmarks.

### Clinical implications

Our findings demonstrate that effective thromboprophylaxis requires more than protocol compliance; it depends equally on drug integrity, mechanical prophylaxis, and vigilant systems monitoring. 79% of confirmed DVT cases were temporally linked to malfunctioning refrigeration of enoxaparin. The pharmacological efficacy of LMWH is known to decline with prolonged exposure to elevated temperatures [4], and systemic cold chain breaches have been implicated in vaccine and drug failures worldwide [8]. This provides new evidence underscoring the critical role of drug storage integrity as a systems-level determinant of surgical patient safety.

This audit represents one of the first documented instances where such a failure was strongly associated with postoperative thromboembolic complications. While cold chain malfunction was the most plausible driver, venous thromboembolism risk is inherently multifactorial. Patients who developed DVT displayed known clinical risk trends, including being older, more likely to undergo emergency surgery, and less likely to receive mechanical prophylaxis. These non-significantly different characteristics highlight the fact that the failure of the cold chain system critically undermined thromboprophylaxis efficacy for patients who were already at high base line VTE risk. Furthermore, two confirmed cases occurred in patients receiving LMWH from unaffected refrigerators, underscoring that individual patient-specific comorbidities and operative factors also contributed to prophylaxis failure.

Comparative analysis of patient characteristics (Table 2) demonstrated that patients with DVT tended to be older, more likely to undergo emergency procedures, and less frequently received mechanical prophylaxis. Although these trends did not reach statistical significance, they highlight important potential confounders. Two cases also occurred in patients whose prophylaxis originated from unaffected refrigerators, underscoring the multifactorial nature of VTE risk, including individual comorbidities and perioperative complications. Contact with the drug manufacturer was not feasible during the audit, and no retained samples were available for laboratory assay, limiting definitive attribution of drug degradation. Nevertheless, the strong temporal and statistical association between cold chain malfunction and DVT incidence points to a significant systems-based threat to patient safety.

### Limitations

Several limitations must be acknowledged. First, four patients with clinically suspected DVT did not undergo confirmatory Doppler ultrasonography, leaving their outcomes uncertain. Although Wells Score was applied uniformly to define suspected cases, we did not compare Wells Scores between confirmed and unconfirmed patients, which could have provided further diagnostic insight. Second, no departmental baseline audit was available, restricting internal comparison with prior institutional experience. Third, pharmacological testing of LMWH batches and manufacturer verification were not performed, preventing direct confirmation of drug degradation. Fourth, the audit did not systematically record bleeding complications, limiting assessment of the overall safety profile of thromboprophylaxis. Fifth, detailed information on other postoperative complications (e.g., anastomotic leak, reoperation, or ICU admission) was unavailable, and the timeline of DVT diagnosis was not captured with sufficient granularity to differentiate inpatient-acquired from post-discharge events. Finally, the single-center, retrospective design limits generalisability.

These limitations are offset by several strengths: systematic use of Doppler confirmation, integration of pharmacy and biomedical engineering data, and the immediate translation of findings into quality-improvement measures. The limited number of DVT events precluded robust multivariable regression analysis; therefore, observed associations should be interpreted with caution. We were also unable to report median timing of inpatient diagnoses, which would have provided additional correlation with perioperative LMWH exposure.

### Future directions

The audit cycle remains open. Corrective measures have already been implemented, including replacement of faulty refrigerators with alarm-equipped units, reinforcement of cold chain monitoring protocols, and improved compliance with mechanical prophylaxis. A prospective re-audit is scheduled for August 2025 to assess the impact of these interventions. Future work should incorporate prospective monitoring, systematic capture of both efficacy outcomes (DVT/PE incidence) and safety outcomes (major and non-major bleeding complications), and stratification of outcomes by inpatient versus post-discharge timing. Integration of validated clinical risk scores such as the Wells Score [5], alongside pharmacological testing of retained drug samples where feasible, will strengthen causal inference. Larger multicenter collaborations may further clarify the interplay between infrastructure reliability and pharmacological prophylaxis outcomes.

This audit demonstrates that infrastructure failures, such as cold chain malfunction, can critically undermine the effectiveness of pharmacological thromboprophylaxis despite adherence to international guidelines. The resulting rise in postoperative DVT highlights the importance of safeguarding drug integrity as a fundamental element of surgical safety. Our findings emphasize that thromboprophylaxis is not only a matter of prescribing the correct regimen, but also ensuring system-level reliability in drug storage and delivery. Following corrective interventions, a re-audit is planned to confirm improvement and sustain quality of care. Broader recognition of cold chain reliability as a patient safety priority may prevent similar events in other resource-constrained surgical settings.

## Recommendations and action plan

Based on our findings, the following corrective measures were implemented:

1. Immediate Replacement of Faulty Refrigerator
  A new temperature-controlled medication fridge with real-time digital monitoring and alarm systems was installed in June 2025.
2. Cold Chain Monitoring Protocol 
  Daily manual temperature checks were reinstated, alongside biweekly pharmacy audits and digital data logging.
3. Staff Education 
  Nursing and pharmacy staff were re-educated on the importance of maintaining cold chain integrity for LMWH and other temperature-sensitive medications.
4. Redesign of Pharmacy-Engineering Workflow 
  A new system for prompt reporting and repair of refrigeration failures was introduced, ensuring maintenance is notified within 12 h of temperature deviation.
5. Audit Expansion and Re-Audit Plan
  A formal re-audit has been scheduled for August 2025 to assess the impact of implemented measures on postoperative DVT incidence and validate restoration of LMWH efficacy under verified cold chain conditions.

## Conclusion

Despite adherence to international prophylaxis guidelines, our surgical ward experienced a significantly elevated postoperative DVT rate. Audit findings identified cold chain malfunction as the most plausible driver of pharmacological prophylaxis failure, compounded by suboptimal mechanical prophylaxis. This demonstrates that safeguarding medication integrity is as crucial as protocol compliance. Corrective measures, including replacement of refrigerators and reinforcement of mechanical prophylaxis use, have been implemented, and a re-audit is planned. Ensuring drug quality through robust infrastructure monitoring should be recognised as a fundamental component of surgical safety worldwide.

## Data Availability

The datasets generated and/or analysed during the current study are available from the corresponding author on reasonable request.
